# Sleep and cognitive aging in the eighth decade of life

**DOI:** 10.1093/sleep/zsz019

**Published:** 2019-01-21

**Authors:** Simon R Cox, Stuart J Ritchie, Mike Allerhand, Saskia P Hagenaars, Ratko Radakovic, David P Breen, Gail Davies, Renata L Riha, Sarah E Harris, John M Starr, Ian J Deary

**Affiliations:** 1 Centre for Cognitive Ageing and Cognitive Epidemiology, University of Edinburgh, Edinburgh, UK; 2 Department of Psychology, University of Edinburgh, Edinburgh, UK; 3 Division of Psychiatry, University of Edinburgh, Edinburgh, UK; 4 Social, Genetic and Developmental Psychiatry Centre, Institute of Psychiatry, Psychology and Neuroscience, King’s College London, London, UK; 5 Faculty of Medical and Health Sciences, University of East Anglia, Norwich, UK; 6 Alzheimer Scotland Dementia Research Centre, University of Edinburgh, Edinburgh, UK; 7 Centre for Clinical Brain Sciences, University of Edinburgh, Edinburgh, UK; 8 Anne Rowling Regenerative Neurology Clinic, University of Edinburgh, Edinburgh, UK; 9 Usher Institute of Population Health Sciences and Informatics, University of Edinburgh, Edinburgh, UK; 10 Department of Sleep Medicine, Royal Infirmary of Edinburgh, Edinburgh, UK; 11 Centre for Genomic and Experimental Medicine, University of Edinburgh, Edinburgh, UK

**Keywords:** daytime sleep, cognitive aging, polygenic scores

## Abstract

We examined associations between self-reported sleep measures and cognitive level and change (age 70–76 years) in a longitudinal, same-year-of-birth cohort study (baseline *N* = 1091; longitudinal *N* = 664). We also leveraged GWAS summary data to ascertain whether polygenic scores (PGS) of chronotype and sleep duration related to self-reported sleep, and to cognitive level and change. Shorter sleep latency was associated with significantly higher levels of visuospatial ability, processing speed, and verbal memory (β ≥ |0.184|, *SE* ≤ 0.075, *p* ≤ 0.003). Longer daytime sleep duration was significantly associated slower processing speed (β = −0.085, *SE* = 0.027, *p* = 0.001), and with steeper 6-year decline in visuospatial reasoning (β = −0.009, *SE* = 0.003, *p* = 0.008), and processing speed (β = −0.009, *SE* = 0.002, *p* < 0.001). Only longitudinal associations between longer daytime sleeping and steeper cognitive declines survived correction for important health covariates and false discovery rate (FDR). PGS of chronotype and sleep duration were nominally associated with specific self-reported sleep characteristics for most SNP thresholds (standardized β range = |0.123 to 0.082|, *p* range = 0.003 to 0.046), but neither PGS predicted cognitive level or change following FDR. Daytime sleep duration is a potentially important correlate of cognitive decline in visuospatial reasoning and processing speed in older age, whereas cross-sectional associations are partially confounded by important health factors. A genetic propensity toward morningness and sleep duration were weakly, but consistently, related to self-reported sleep characteristics, and did not relate to cognitive level or change.

**Statement of Significance**

Using a large cohort of older participants and advanced statistical modeling of longitudinal data at ages 70, 73, and 76, we find that longer daytime sleeping is associated with steeper declines in visuospatial reasoning and processing speed. These findings were robust to correction for important health covariates and multiple comparisons. Whereas polygenic scores (PGS) for chronotype and sleep duration were associated with relevant self-reported sleep measures, neither PGS predicted level or change in cognitive functioning. A greater understanding of a possible causal relationship between daytime sleeping and cognitive function (and its direction) are a priority for future study.

## Introduction

Advancing age is associated with complex changes in sleep patterns and to increasing risk of cognitive decline. Older individuals exhibit shorter sleep time, a lower percentage of rapid eye movement, a longer relative sleep latency, with an increase in the proportion of lighter sleep stages 1 and 2 and reductions in the deeper sleep at stages 3 and 4 [1]. Alterations in circadian regulation also lead to advanced timing of sleep to earlier hours and difficulties with sleep consolidation [2]. Excessive daytime sleepiness also increases with age, particularly with respect to the propensity for actually falling asleep in the daytime [3–5]. Overall mean declines in multiple domains of cognitive function are relatively well characterized. With the exception of crystallized intelligence, most complex cognitive processes show some degree of mean decline into older age, though estimates differ with respect to the age at which decline begins, the composition of cognitive domains, and their trajectories of decline [6–8]. Nevertheless, there are substantial individual differences in aging-related sleep characteristics, and in the degree to which generally healthy older individuals experience cognitive decline.

In the short-term, suboptimal sleep—mainly examined using acute sleep restriction in tightly controlled laboratory environments—is detrimental to cognitive functioning [9–11]. Sleep restriction may contribute to the accumulation of amyloid-β due to region-specific synaptic activity [12–15]. and because it interferes with the cerebral clearance of neurotoxic waste products (including amyloid-β [16–18]). Sleep dysfunction—measured in observational settings—is common in mild cognitive impairment and dementia and may be reflective of underlying neurodegeneration [19, 20], leading to interest in whether (and which) sleep characteristics denote poorer and declining cognitive functioning in the healthy aging over the longer-term. It remains unclear whether sleep disturbance is a useful marker for, or risk factor of, concomitant age-related brain and cognitive decline. A better understanding of sleep-cognitive associations will aid identification of at-risk groups and future development of therapies to ameliorate cognitive decline [21].

Longitudinal epidemiological investigations into sleep and cognitive aging remain comparatively scarce [22, 23]. Recent reviews [23, 24] including both cross-sectional and longitudinal studies suggest that the most consistent associations with poorer cognitive ability are sleep duration (see meta-analyses [23, 25]), and excessive daytime sleepiness—often including longer daytime sleeping ([26–32], but see [22]). In contrast, a large cross-sectional study found that daytime napping >60 minutes was associated with significantly higher Mini-Mental State Examination (MMSE) among those with a morning chronotype when compared to non-nappers, though this was not true among evening or intermediate type elderly adults [33]. Poorer self-reported sleep quality has also been linked with poorer cognitive function in several studies [34–37].

Nevertheless, there remains a great deal of ambiguity regarding many of these findings given the methodological heterogeneity across studies [23, 24]. Factors include variable and sometimes brief follow-up periods where longitudinal data are available, and a widespread absence of correction for multiple comparisons, leading to a higher likelihood of reporting false-positives. Age groups are differentially represented across studies, which could obscure consistent detection of sleep characteristics that are more or less important for cognitive functioning during different epochs of adulthood. Moreover, a variety of approaches are used to measure sleep and cognitive function. For example, global measures such as MMSE or dichotomous outcomes of cognitive decline are often employed, which limits statistical power to detect associations which may exist along a continuum [23, 24], and such coarse-grained clinical measurements often exhibit ceiling effects in the general non-pathological aging population. It is important to provide robust and accurate measures of cognitive function across different domains since they may be differentially affected by sleep [23, 36]. Finally, sleep and poorer cognitive function could show significant associations because both are influenced by factors such as depression, pain levels and other health conditions including diabetes and cardiovascular disease, though correction for such measures is not applied routinely [36].

Genetic influences on sleep characteristics may also be relevant to differences in cognitive aging. Data from family and twin studies suggest that the heritability of chronotype (the two extremes of which are known as “morningness” and “eveningness”) is ~50% (reviewed in [38]), and that of sleep duration is ~31–65% in adults [39–41]. The use of large-scale Genome-Wide Association Studies (GWAS) to identify the molecular genetic contributions to sleep duration and chronotype among unrelated individuals identified loci containing genes with a known role in circadian regulation [42–45]. Importantly, the summary statistics from a GWAS (which denote the relative importance of all genetic loci for a phenotype of interest) can be used to create a polygenic score (PGS) [46] of that same phenotype in an entirely separate sample, resulting in a PGS for each individual. One advantage of this approach is that principal driver of prediction accuracy is the size and quality of the original GWAS [47], making this an appropriate approach for prediction into other, smaller, samples (provided those samples are still adequately powered to detect relatively modest effect sizes). In the context of sleep and cognitive aging, this affords the opportunity to test the hypothesis that genetic propensity for chronotype or sleep duration is related to relevant sleep characteristics, and also individual differences in cognitive aging.

In the current study, we investigated whether there were significant associations between poorer sleep (measured via self-report and genetic liability scores) and poorer cognitive functioning (lower level and steeper decline) over 6 years in a large cohort study of community-dwelling older adults, all born in 1936. We derived three domains of cognitive function (visuospatial reasoning, processing speed, and memory) from an extensive battery of validated cognitive tests, and accounted for important health factors. We then leveraged summary results of large independent GWAS analyses to test associations of PGS for chronotype and sleep duration with self-reported sleep phenotypes, and with cognitive aging differences.

## Methods

### Participants

Data were drawn from the Lothian Birth Cohort 1936 (LBC1936 [48–51]). Surviving participants of the Scottish Mental Survey of 1947—all born in 1936—most of whom were resident in the Edinburgh and Lothians area of Scotland, were recruited into the LBC1936 at Wave 1 (*N* = 1091, aged ~70 years). Cognitive, health and physical function were assessed between 2004 and 2007, including self-reported years of education (M = 10.75, SD = 1.13). Participants were subsequently assessed triennially at Wave 2 (*N* = 866, from 2007 to 2011) and at Wave 3 (*N* = 697, from 2011 to 2013) at ages ~73 and ~76, respectively. When contacted to arrange an appointment, participants were able to choose from a range of appointment start times (typically between 9 am and 11 am); whereas we did not systematically control time since waking, this would have allowed some compatibility with individual differences in chronotype.

### Medical assessments

Among other assessments, a medical interview at each wave collected information on cardiovascular disease history and arthritis, and whether they had received a diagnosis of diabetes, hypertension, and dementia from a medical practitioner. Height and weight were measured from which body mass index (BMI) was calculated. Participants completed the Hospital Anxiety and Depression Scale (HADS [52]) and the MMSE [53] at each wave, as an indicator of possible pathological cognitive aging.

### Sleep

At wave 3 (age ~76), participants also completed a questionnaire in which they were asked to rate various aspects of their sleep, with reference to weekdays and weekends separately. From their responses, we obtained six variables of interest: sleep quality, bedtime, latency, wake time, nighttime sleep duration and daytime sleep duration. Sleep quality was assessed with the question “During the past month, how would you rate your sleep quality overall?” from 0 (very bad) to 3 (very good), taken from the Pittsburgh Sleep Quality Index [54]. Based on the Patient-Partner Questionnaire used in the Respiratory Medicine Unit, Royal Infirmary of Edinburgh (author R.L.R.), participants were also asked to provide estimates for their bedtime (“What time do you usually go to bed at night?”), sleep latency (“How long does it take you to fall asleep at night?”), wake time (“What time do you usually get up in the morning?”), as well as sleep duration at night (“How many hours of actual sleep do you usually get at night?”) and during the day (“How long do you usually spend asleep during the day and evening?”).

### Cognitive testing

We fitted structural equation models to investigate cognitive abilities at the domain level using test scores from waves 1–3 (see Statistical Analysis). Categorization of the individual subtests into cognitive domains was as follows, consistent with prior work on the correlational structure of the cognitive test battery in the LBC1936 study [55, 56]:

Visuospatial ability comprised Matrix Reasoning and Block Design from the Wechsler Adult Intelligence Scale 3rd UK Edition (WAIS-III-UK [57]), and Spatial Span (sum of forward and backward) from the Wechsler Memory Scale, 3rd UK Edition (WMS-III-UK [58]).

Processing Speed included the Symbol Search and Digit-Symbol Substitution tests from the WAIS-III-UK. It also used a measure of Four-Choice Reaction Time [59] and Visual Inspection Time [60]. In the former, participants were presented with a number (1–4) in each of 40 trials, and have to press a corresponding response key as quickly as possible. The outcome is the average response time overall correct responses. In the latter, the goal is to identify which of two possible (markedly different, and backward-masked) figures they have been presented within each trial. There is no response time limit, but the presentation time varies across 15 increments (10 trials each) from 6 to 200 ms. The outcome measure is the total number of correct responses.

Verbal Memory ability was derived from Logical Memory (sum of immediate and delayed), and Verbal Paired Associates (sum of immediate and delayed) from the WMS-III-UK, and the Digit Span Backward subtest from the WAIS-III-UK.

### PGS for chronotype and sleep duration

Using venous blood drawn from the LBC1936 participants, DNA was extracted and genotyping was conducted using the Illumina 610-Quadv1 whole-genome SNP array (San Diego, CA) at the Wellcome Trust Clinical Research Facility, Western General Hospital, Edinburgh. We used summary data of a recent GWAS of chronotype (morningness: “Definitely a ‘morning’ person”, “More a ‘morning’ than ‘evening’ person”, “More an ‘evening’ than a ‘morning’ person”, “Definitely an ‘evening’ person” or “Do not know”) and sleep duration (an estimate of the average number of hours slept in a 24-hour period) in 128 266 individuals [44]. From these, we created PGS in all genotyped LBC1936 participants using PRSice software [61]. PRSice calculates the sum of alleles associated with the phenotype of interest across many genetic loci, weighted by their effect sizes estimated from a GWAS of the corresponding phenotype in an independent sample. Clumping was used to obtain SNPs in linkage disequilibrium with an *r*^2^<0.25 within a 250 kb window. SNPs used to create the PGS were selected according to the significance of their association with the phenotype at the following significance thresholds: 0.01, 0.05, 0.1, 0.5, and 1, where the latter denotes the use of all SNPs. Four multidimensional scaling (MDS) components were obtained as the first four components of a principal components analysis of all SNPs, and were included as covariates in subsequent PGS analyses to account for any possible effects of population stratification among this white Scottish sample (see next section).

### Statistical analysis

Participants with self-reported history of dementia or an MMSE score of <24 (at any wave) were removed prior to analyses. For the sleep data, in instances where two values were entered (e.g. what time do you fall asleep = “2200–2230”), the average value was used. Nonnumerical responses for numerical fields (e.g. how long does it take you to fall asleep—“not long”) were not included. Ratings of how quickly participants fell asleep and how long they slept during the day were log transformed to correct skewness. Daytime sleep duration (reported in minutes) was converted to hours prior to entry into the models. Weekend and weekday measures were highly correlated in all instances (*r* > .864, *p* < .001), and so average values were used in subsequent analyses.

**Figure 1.**
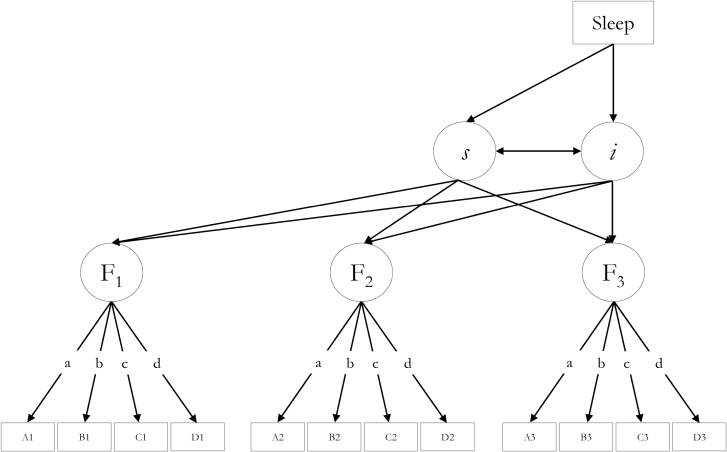
A schematic latent growth curve model, in which self-reported sleep is associated with the intercept and linear slope of a latent factor of cognitive function (F) at wave *j* based on test scores A_j_, B_j_ …, whose intercepts, and factor loadings (a–d) on the latent cognitive factor are constrained to equality across the three waves. Residual correlations between the same tests across waves were allowed, and manifest cognitive variables were corrected for sex and mean-centered age_j_ within the model (paths not shown—see Statistical Analysis). The model was centered on wave 3 (age 76) when sleep data were collected. The regressions of Sleep (predictor) on cognitive intercept (i) and slope (s) were the associations of interest.

We used structural equation modeling to examine associations between sleep indices (self-reported and PGS) and both the level and change in each domain of cognitive, function (visuospatial reasoning, processing speed, memory). Specifically, we employed latent growth curve *SEM*; for latent cognitive factors, we fitted a separate “curves of factors” model [62] (Figure 1) for each cognitive domain and each sleep measure. In these instances, we imposed factorial invariance, constraining the intercepts of each cognitive test and their loadings on the latent variable to equality across waves. To allow the model to converge upon within-bounds estimates, we set some residual variances to zero (factor 1 in the visuospatial and processing speed models). In order to reduce bias due to missingness, we employed full-information maximum likelihood based on the assumption of Missing At Random (MAR [63]).

Before entry into the model, sleep measures were corrected (residualized using linear regression) for age in days and sex. Within the *SEM*, each cognitive test was corrected for sex and age in days at data collection; mean age at each wave was centered on zero to remove intra-wave age variance while preserving the inter-wave differences in cognitive performance. The models were centered on wave 3 (when sleep data were gathered), such that associations with sleep and cognitive intercept represented level-level associations at wave 3 (age ~76 years). Associations between sleep and cognitive slopes, therefore, indicated whether those with particular self-reported sleep characteristics had been experiencing more or less cognitive decline over the preceding 6 years. To account for the possible influence of health factors on sleep and cognitive decline metrics, we re-ran models with initially significant (FDR *q* < 0.05) sleep-cognitive associations, this time correcting sleep and cognitive ability for time-varying measures of anxiety and depression symptoms (HADS), BMI, hypertension, diabetes, cardiovascular history, and arthritis.

Given the arbitrary scale of latent factors, we also conducted post-hoc analyses to aid interpretation of the reported associations between cognitive slope and daytime sleep duration. We fit two separate models in which we correlated the slope from a growth curve for Digit-Symbol Substitution subtest (a well-known test of processing speed), with daytime sleep, correcting for age, sex and health, as above. During the review process, it was proposed to additionally test the effect of correcting sleep latency and cognitive function intercept for years of education (as a marker of cognitive reserve), instead of using health covariates. This was done in order to test a hypothesis of reverse causation, whereby cognitive reserve might explain the attenuation of associations between sleep latency and cognitive level by health covariates. In such circumstances, years of education would account for approximately the same (or greater) proportion of latency-cognitive associations as did health.

Finally, we examined the association between self-reported sleep and PGS for chronotype and sleep duration using linear regression, and subsequently tested whether these PGS were associated with the level and change of the cognitive domains (using the same *SEM* growth curve framework as above). In both analyses, the four MDS components were included as covariates—note that the GWAS from which the PGS were obtained [43] already controlled for age and sex, and so these were not included as covariates here. We presented PGS and sleep measures across all PGS thresholds for illustrative purposes. However, as suggested previously [64] we restricted our primary analyses (associations between PGS and cognitive abilities) to PGS created at *p* ≤ 1 (the most inclusive threshold) in order to maximize the potential predictive capacity of studies and reduce the degree of redundant hypothesis testing among highly collinear metrics.

All analyses were conducted in R v 3.2.2 (“Fire Safety” [65]). *SEM* was conducted with the “*lavaan*” package [66] and the resultant *p*-values for the associations of interest (see asterisks in Figure 1) were corrected for multiple comparisons with false discovery rate (FDR [67]) using the “*p.adjust*” function in R.

## Results

### Relationships among sleep characteristics

Participant characteristics across waves are reported in Supplementary Table S1, and individual cognitive test trajectories across this period have been previously reported [55]. Sleep characteristics are reported in Tables 1 and 2. Participants who reported better sleep quality were, on average, those who took less time to fall asleep (ρ = −0.397, *p* < 0.001), slept for longer during the night (*r* = 0.530, *p* < 0.001), woke up later (ρ = 0.093, *p* = 0.017) and slept less during the day (*r* = −0.091, *p* = 0.023). As well has having poorer sleep quality, individuals who slept for longer during the day reported sleeping less at night (*r* = −0.166, *p* < 0.001), driven by a later bedtime (*r* = 0.106, *p* = 0.008), earlier wake time (*r* = −0.083, *p* = 0.038), but not a longer latency (*r* = 0.012, *p* = 0.782). More sleep at night was associated with a shorter latency (*r* = −0.304, *p* < 0.001) an earlier bedtime (*r* = −0.096, *p* = 0.015) and a later wake time (*r* = 0.213, *p* < 0.001).

**Table 1.** Self-reported sleep characteristics, collected at wave 3

	Units	Mean/median	SD/IQR	*N*
Quality	0–3	2.00	1.00	664
Bedtime	24-hour clock	23:00	52.09 minutes	656
Latency^a^	Minutes	20	20	573
Wake time	24-hour clock	07:52	53.40 minutes	654
Length night	Hours, minutes	6 hours 54 minutes	1 hour 17 minutes	653
Length day^a^	Minutes	10	37.5	629

^a^Median and interquartile range provided for variables that are subsequently log transformed prior to analysis, and for sleep Quality (ordinal).

**Table 2.** Associations among self-reported sleep variables

	Quality	Bedtime	Latency	Wake time	Length night	Length day
Quality	*****	0.085	−0.397	0.093	0.530	−0.091
Bedtime	0.029	*****	−0.142	0.341	−0.096	0.106
Latency^a^	<0.001	0.001	*****	0.044	−0.304	0.012
Wake time	0.017	<0.001	0.292	*****	0.213	−0.083
Length night	<0.001	0.015	<0.001	<0.001	*****	−0.166
Length day^a^	0.023	0.008	0.782	0.038	<0.001	*****

Pearson’s *r* (upper diagonal) and *p*-values (lower diagonal) are reported, except for associations with sleep quality (ordinal), for which Spearman’s ρ is used.

### Associations between self-reported sleep and cognitive measures

Unstandardized coefficients, standard errors and *p*-values of the associations between sleep measures and the level and change in cognitive functioning domains are reported in Table 3. Model fit indices are shown in Supplementary Tables S2–S4. All models exhibited a good fit to the data.

**Table 3.** Self-reported sleep predictors of visuospatial ability level at age 76, and change from 70 to 76 years

	Intercept			Slope		
	Est.	*SE*	*p*	Est.	*SE*	*p*
Visuospatial reasoning						
Sleep quality	0.074	0.084	0.377	0.004	0.008	0.668
Bedtime	0.155	0.073	0.033	0.001	0.007	0.879
Latency^a^	−**0.215**	**0.072**	**0.003**	0.002	0.007	0.790
Wake time	−0.020	0.070	0.777	0.005	0.007	0.500
Length night	0.031	0.048	0.517	0.001	0.005	0.856
Length day^a^	−**0.533**	**0.197**	**0.007**	−**0.071**^**b**^	**0.020**	**<0.001**
Speed						
Sleep Quality	0.055	0.065	0.400	0.000	0.006	0.977
Bedtime	0.052	0.056	0.349	−0.007	0.005	0.205
Latency^a^	−**0.206**	**0.075**	**0.006**	−0.005	0.009	0.586
Wake time	−0.030	0.054	0.575	−0.004	0.005	0.455
Length night	0.015	0.037	0.688	0.002	0.003	0.563
Length day^a^	−**0.495**	**0.151**	**0.001**	−**0.062**^**b**^	**0.014**	**<0.001**
Memory						
Sleep quality	0.048	0.087	0.584	−0.013	0.011	0.218
Bedtime	0.161	0.077	0.036	0.019	0.009	0.038
Latency^a^	−**0.221**	**0.075**	**0.003**	−0.004	0.009	0.635
Wake time	−0.089	0.074	0.226	−0.007	0.009	0.449
Length night	−0.115	0.051	0.026	−0.012	0.006	0.057
Length day^a^	−0.263	0.212	0.215	−0.028	0.026	0.269

Unstandardized coefficients (Est.) and standard errors (*SE*) from latent growth curve models are reported. Bold typeface denotes *q* < 0.05.

^a^Log transformed.

^b^Association remains FDR-significant following further correction for health measures.

For all cognitive domains, there was a significant cross-sectional association between cognitive ability at age 76 (visuospatial, processing speed and memory) and sleep latency. Individuals who reported taking less time to fall asleep at night had significantly better visuospatial ability (β = −0.215, *SE* = 0.072, *p* = 0.003), processing speed (β = −0.206, *SE* = 0.075, *p* = 0.006), and verbal memory (β = −0.221, *SE* = 0.075, *p* = 0.003) at age 76. Each survived FDR correction. There were no significant associations between sleep latency and 6-year change in any of the cognitive factors (*p* ≥ 0.635).

Individuals who reported sleeping for longer during the day exhibited significantly lower visuospatial ability (β = −0.533, *SE* = 0.197, *p* = 0.007) and slower processing speed at age 76 (β = −0.495, *SE* = 0.151, *p* = 0.001), and steeper 6-year decline in terms of visuospatial reasoning (β = −0.071, *SE* = 0.020, *p* < 0.001), and processing speed (β = −0.062, *SE* = 0.014, *p* < 0.001), but not verbal memory ability (β = −0.028, *SE* = 0.026, *p* = 0.269). No other associations between self-reported sleep metrics and cognitive level or change survived FDR correction.

**Figure 2.**
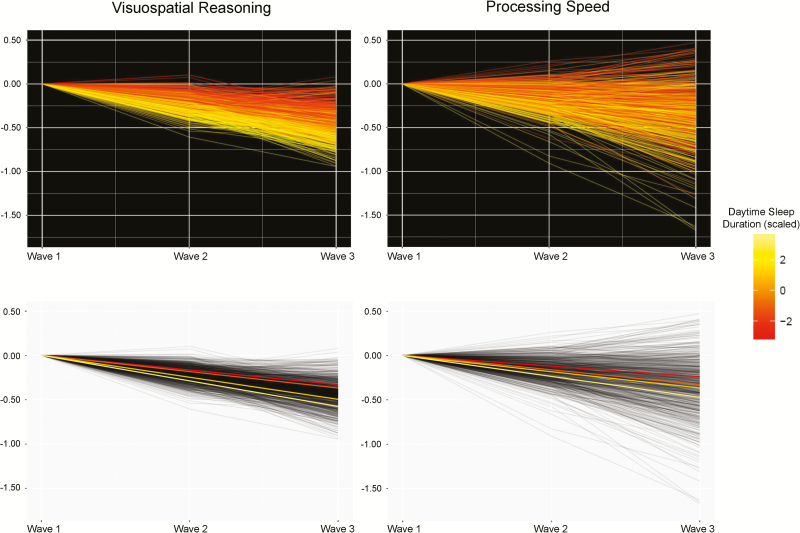
Significant associations between greater daytime sleep duration at age 76 and 6-year cognitive decline. Cognitive factor scores at each wave were extracted from the structural equation models in which both outcome and predictor variables are corrected for sex, intra-wave age variation, and time-varying health measures (HADS, BMI, hypertension, diabetes, cardiovascular history, and arthritis), and were normalized for wave 1 score to illustrate individual differences in trajectories of change. Top row: individual cognitive trajectories as modeled (continuous), and are shaded to indicate less-more (red-yellow) daytime sleep duration. Bottom row: regression lines for four equally sized groups of daytime sleep duration in the same data, for illustration purposes only.

For the instances of FDR-corrected significant sleep-cognitive associations, we subsequently corrected the sleep and cognitive data for important health covariates (HADS, BMI, hypertension, diabetes, cardiovascular history, and arthritis). Health-corrected associations for longer sleep during the day remained FDR-significant for greater decline in visuospatial ability (β = −0.072, *SE* = 0.020, *p* < 0.001) and processing speed (β = −0.056, *SE* = 0.014, *p* < 0.001). The associations between duration of sleep during the day and cognitive domains are illustrated in Figure 2. As an indication of the magnitude of the association in test score terms, a 50% increase in hours of daytime sleep duration represented a decline of ~1.3 points on Digit-Symbol Substitution test from age 70 to 76, holding age, sex and health measures equal (Supplementary Table S5). The cross-sectional association between daytime sleep duration and the intercepts for visuospatial (β = −0.098, *SE* = 0.073, *p* = 0.180) and processing speed (β = −0.298, *SE* = 0.157, *p* = 0.058) were nonsignificant.

Though effect sizes were only partially attenuated, associations between sleep latency and the intercepts of visuospatial (β = −0.098, *SE* = 0.073, *p* = 0.180; 54% attenuation), processing speed (β = −0.100, *SE* = 0.058, *p* = 0.086; 51%), and memory (β = −0.163, *SE* = 0.078, *p* = 0.037; 26%) were all FDR nonsignificant when including health covariates. Given the possible reverse-causation interpretation of this result (see Methods and Discussion), we also investigated the attenuation of these latency-cognitive associations by years of education (as a measure of cognitive reserve; simple bivariate analyses revealed that years of education was modestly and nonsignificantly associated with lower sleep latency *r* = −0.078, *p* = 0.062, and significantly negatively associated with HADS *r* = −0.123, *p* = 0.001, diabetes *r* = −0.088, *p* = 0.023 and BMI *r* = −0.111, *p* = 0.004 at wave 3). There was comparatively less attenuation of the latency-cognitive level associations when correcting cognitive level and sleep latency for years of education, instead of health covariates for visuospatial (β = −0.158, *SE* = 0.069, *p* = 0.021, 27% attenuation) and processing speed (β = −0.154, *SE* = 0.055, *p* = 0.005, 25%) though it was not substantially different for memory (β = −0.156, *SE* = 0.077, *p* = 0.041, 29% attenuation).

### Associations between self-reported sleep and PGS for chronotype and sleep duration

**Figure 3.**
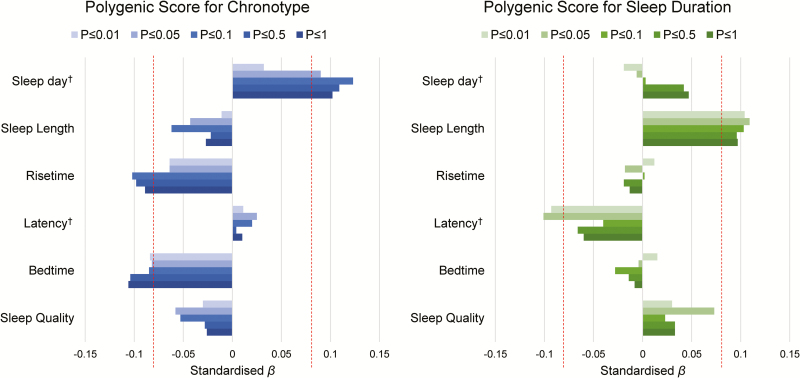
Associations between self-reported sleep characteristics and polygenic scores for chronotype (left) and sleep duration (right) at all thresholds. Red vertical dashed lines are indicative of nominal significance (*p* < 0.05). None survived FDR correction. Magnitude of associations (*x*-axis) are standardized regression coefficients, controlling for the four MDS components.

Associations between polygenic and phenotypic sleep indices are reported in Figure 3 and Supplementary Tables S6 and S7. Participants with a higher PGS for chronotype (greater “morningness”) reported going to bed earlier (all thresholds, standardized β range = −0.106 to −0.082, *p* range = 0.009 to 0.046), rising earlier (at thresholds *p* ≤ 1, 0.5 and 0.1: standardized β range = −0.102 to −0.089, *p* range = 0.013 to 0.031) and sleeping for longer during the day (all thresholds except *p* ≤ 0.01: standardized β range = 0.123 to 0.090, *p* range = 0.003 to 0.030). These were all nominally significant (*p* < 0.05), but did not survive FDR correction.

A greater PGS for longer sleep duration was associated with longer self-reported sleep during the night (all thresholds, standardized β range = 0.096 to 0.109, *p* range = 0.010 to 0.025), and less time taken to fall asleep (at threshold *p* ≤ 0.05, β = −0.101, *p* = 0.027; and at *p* ≤ 0.01, β = −0.093, *p* = 0.043). None of these associations survived correction for multiple comparisons.

Associations between cognitive abilities (level and change) and PGS (chronotype and sleep duration at *p* ≤ 1) are shown in Table 4. There was a nominally significant association between a lower PGS for sleep duration and decline in visuospatial ability (β = −0.013, *SE* = 0.006, *p* = 0.049); no associations were significant following FDR correction.

**Table 4.** Polygenic score predictors (*p* ≤ 1) of cognitive ability level at age 76, and change from 70 to 76 years

	Intercept				Slope		
	Estimate	*SE*	*p*	Estimate	*SE*	*p*
Chronotype						
Visuospatial	0.016	0.054	0.764	−0.003	0.006	0.617
Speed	−0.016	0.041	0.694	−0.005	0.004	0.214
Memory	−0.024	0.057	0.679	−0.008	0.008	0.319
Duration						
Visuospatial	0.029	0.055	0.597	−0.012	0.006	0.049
Speed	0.057	0.042	0.174	−0.000	0.004	0.988
Memory	0.072	0.059	0.224	0.006	0.008	0.456

Unstandardized estimates from latent growth curve models are reported. No associations survived FDR correction.

## Discussion

In this longitudinal study of generally healthy, community-dwelling older adults, individuals reporting a longer sleep latency showed significantly poorer (cross-sectional) levels of visuospatial reasoning, processing speed, and verbal memory. Those who reported sleeping longer during the day and evening also exhibited significantly lower processing speed, and steeper longitudinal declines in visuospatial reasoning and processing speed ability over the preceding 6-year period. All cross-sectional associations were attenuated to nonsignificance when additionally controlling for health status. However, associations between daytime sleep duration and declining visuospatial reasoning and processing speed abilities from 70 to 76 years remained significant once both cognitive and sleep variables had been corrected for depressive symptoms, BMI, hypertension, diabetes, cardiovascular history, and arthritis. Finally, we found that higher PGS of chronotype and sleep duration, derived from a large GWAS in an independent sample, were associated with corresponding self-reported phenotypes in the LBC1936, but that these scores did not predict cognitive level or change in any domain after FDR correction.

The association between decline in cognitive abilities and length spent sleeping during the day correspond with—and extend—previous studies in samples with wider age ranges that reported associations between excessive daytime sleepiness (including daytime napping) and poorer cognitive functioning [25–33]. To the best of our knowledge, the present study is the first to show a relationship between daytime sleep duration and longitudinal decline of processing speed and visuospatial ability in older age. To date, prior work has mainly constituted global cognitive outcomes on cross-sectional [26, 28] and longitudinal [30] data, or detailed cognitive testing but using only cross-sectional data [27]. Tsapanou and colleagues [29] examined longitudinal change in different cognitive domains with sleep characteristics, and found a significant association between greater daytime somnolence and more steeply declining processing speed in a similar-size cohort of similar age (M = 75.3, SD = 6.1 years) over a shorter (3.2 year) follow-up. However, their measure of daytime somnolence also incorporated self-report of daytime drowsiness, difficulty staying awake, and measured whether naps >5 minutes were taken during the day. On the other hand, our findings point to the amount of time spent asleep during the day (measured as a continuous variable) as an important correlate of cognitive decline in the eighth decade of life.

Moreover, we considered important health covariates that confounded the initially significant associations between longer sleep latency and lower levels of cognitive function. This could indicate that these health factors are related to poorer cognitive function because they interfere with the time taken to fall asleep (e.g. discomfort caused by arthritis). However, were this a causal path, we might have expected to find (at least) indicative associations between sleep latency and cognitive *decline* as well as with *level*. An alternative explanation is that of reverse causation [8]. That is: those with higher cognitive function are less likely to suffer from health issues [68], some of which might prolong sleep latency. Thus, general cognitive level might influence health factors that might impact sleep latency, but sleep latency may not be exerting a causal effect on cognitive function. We found little evidence that cognitive reserve (years of education) could account for as much of the associations between sleep latency and cognitive level as could health measures (with the exception of memory), though the presence of some modest attenuation could be indicative that these possible explanations are not mutually exclusive. It is particularly notable that the longitudinal associations between daytime sleep duration and cognitive function were not similarly confounded, and that there were associations between daytime sleep duration and cognitive level as well as change.

We also investigated the prediction of self-reported sleep facets in LBC1936 based on the participants’ genetic information alone, using the summary statistics from a recent large GWAS of chronotype and sleep duration. The PGS for sleep duration was associated—at *p* < 0.05—with self-reported sleep duration at all thresholds in the same direction, though none survived FDR correction. Similarly, PGS for chronotype (being a “morning person”) was nominally related to an earlier bed time and an earlier wake time at the majority of thresholds, as well as with longer daytime sleep duration, though none of these survived correction for multiple comparisons. However, we did not find that these PGS (at the most inclusive threshold: *p* ≤ 1) were associated with cognitive level and change. Whereas the predictive accuracy of PGS is driven primarily by the size of the sample in which the originating GWAS was conducted [47], and that we were adequately powered to detect associations of the magnitude reported in prior work in this cohort (which identified significant associations between PGS for schizophrenia and both cognitive function and brain imaging metrics [69, 70]), we acknowledge that larger, multi-cohort, meta- and mega-analytic strategies (e.g. [71, 72]) will provide additional statistical power to detect even smaller effect sizes with greater reliability. Nevertheless, the genetic findings reported here might suggest that the strategy of analyzing correlates of PGS of sleep characteristics holds promise for quantifying the contribution that a genetic predisposition to particular sleep facets makes to variation in important life outcomes.

Significant associations between ostensibly “poorer” sleep characteristics and cognitive outcomes in later life lend themselves to several potential causal hypotheses. It is possible that the accumulation of a sleep deficit (or debt) occurs with changes in lifestyle from chronic, responsibility-driven sleep patterns (governed by employment, family or social activities, for example) in older age, and is expressed through important outcomes such as cognitive, physical, mental health, and general, multisystem biological dysregulation [73, 74]. With specific reference to our findings, daytime napping may fulfill a variety of needs including the need to rectify a sleep deficit accrued via disrupted nocturnal sleep due to shift work, a change in lifestyle due to retirement or reduction in child-rearing activities, alterations in circadian rhythmicity (leading to earlier onset of sleepiness in the evening) or a reduction in activity levels with the onset of comorbid illnesses such as cardiovascular or metabolic disease [2, 4, 23, 74]. Daytime napping may also be partly driven by the need to redress sleep disruption via conditions such as obstructive sleep apnea; treatment of which can reduce nap frequency and duration [75].

Associations between daytime napping and cognitive decline might also reflect the age-related disruption of brain centers involved in circadian regulation of sleep [76], potentially driven by nascent neurodegenerative diseases, resulting in increased daytime sleep as a consequence, rather than cause, of age-related brain structural decline. In either case, it could disrupt the proposed restorative functions served by sleep, which may be more effective in deeper phases [18]. It is equally possible that individuals’ sleepiness during a cognitive appointment during which no naps were scheduled had an adverse effect on cognitive performance. However, cognitive testing times were flexible to fit with participants’ chronotype, and one might not expect to see an increasing cognitive disadvantage of daytime sleeping across time (though longitudinal sleep measures would be required to fully test this). With respect to daytime sleeping, the potential catalyst may vary with age, meaning that analyses among samples with large age ranges would be partially confounded, especially where health issues were not considered as covariates. Our single-year-of-birth cohort and use of time-varying health covariates could partly militate against such confounding, though our data cannot resolve speculation about the specific needs that daytime napping fulfills. In addition, we were unable to analyze complex interactions such as whether daytime napping of a particular length was differentially important for cognitive decline among different chronotypes [33]. It is possible that a greater understanding of individual differences in the underlying causes of daytime napping might offer further insights into variation in cognitive aging trajectories.

Some other limitations are also noteworthy. Whereas our cognitive data were longitudinal and allowed us to derive latent measures of level and change, our measures of sleep were measured only at a single—most recent—time point. Though these data are correlational (and thus causation could equally not have been inferred from the same associations if the sleep data were collected at the first wave), our data cannot speak to the temporal order or coevolution of sleep and cognitive change. For example, increases in sleep length are reportedly associated with increased risk of all-cause dementia [25], but we were unable to address these questions in our sample of non-demented older adults. Our measures of sleep were self-reported. While laboratory-obtained measures from polysomnography are too burdensome and invasive in the context of this longitudinal aging study, it is important to note that the accuracy of subjective measurements made here are likely to vary as a function of the facet of sleep in question, and with individual differences in participant characteristics. Prior work comparing subjective / self-report sleep measures with objective measurements from actigraphy indicate that measures of sleep quality, latency and duration agree with moderate to large effect sizes among older individuals [77, 78] and in younger adults for sleep onset, wake time and total sleep time [79]. Additionally, individual differences in agreement between objective and subjective measures vary as a function of participant characteristics such as hostility, health, and motivation [78, 79]. The associations we report between these self-reported measures and PGS for sleep duration and chronotype may add some assurance of external validity, but we caution that these self-reported are likely to be a noisier reflection of participants’ sleep characteristics than actigraphy (for example), and will be partly confounded by a number of other factors we did not account for. The generalizability of our findings is limited due to the narrow age range of our entirely white Scottish sample, but the 6 years of follow-up and use of a same-year-of-birth cohort have allowed us to account for the important confounds of age and individual differences in ethnicity. Though we acknowledge that there are many other model specifications that would allow these relationships to be examined at different levels of granularity (e.g. splitting working and episodic memory), we classified the cognitive tests into domains according to their correlational structure (consistent with prior work in this cohort [55, 56]). Finally, the current analyses did not consider variables such as caffeine consumption, medications, and technology use, which may influence sleep hygiene [80, 81] and would benefit from future work in relation to age-related changes in sleep and cognitive function. Information on sleep apnea prevalence were not available here, but are also likely to bear on both sleep and cognitive characteristics [82].

In conclusion, we employed advanced statistical models and important health covariates in a large longitudinal sample, and identified significant associations between greater time spent napping during the day and greater cognitive decline in visuospatial reasoning and processing speed from age 70 to 76 years. We also found that PGS for sleep duration and chronotype were weakly, but consistently, associated with self-reported facets of sleep, but that these PGS did not predict cognitive changes after correction for multiple comparisons. Further work should focus on identifying the cerebral underpinnings of these cognitive changes, and on ascertaining whether individual differences in sleep duration during the day might offer greater power to detect individuals at greatest risk of cognitive decline.

## Funding

The LBC1936 and this research are supported by Age UK (Disconnected Mind project) and by the UK Medical Research Council (MRC; G0701120, G1001245, MR/M013111/1; MR/R024065/1). R.R. is funded by the Motor Neurone Disease Association and Motor Neurone Disease Scotland. This work was undertaken within The University of Edinburgh Centre for Cognitive Ageing and Cognitive Epidemiology (www.ccace.ed.ac.uk), funded by the MRC and the Biotechnology and Biological Sciences Research Council (MR/K026992/1). This report represents independent research part-funded by the National Institute for Health Research (NIHR) Biomedical Research Centre at South London and Maudsley NHS Foundation Trust and King’s College London. The views expressed are those of the author(s) and not necessarily those of the NHS, the NIHR or the Department of Health.

*Conflict of interest statement*. None declared.

## Supplementary Material

Supplementary Materials
