# Supplementary material for: Sleep and cognitive aging in the eighth decade of life
Source: Sleep. 2019 Jan 21;42(4):zsz019. doi: 10.1093/sleep/zsz019 (PMC6448287; doi:10.1093/sleep/zsz019)
Supplement: Supplementary Materials [file zsz019_suppl_supplementary_materials.docx]

**Supplementary Materials: Sleep and cognitive ageing in the 8^th^ decade of life.**

Simon R. Cox, Stuart J. Ritchie, Michael Allerhand, Saskia P. Hagenaars, Ratko Radakovic, David P. Breen, Gail Davies, Renata L. Riha, Sarah E. Harris, John M. Starr, Ian J. Deary.

*Table S1.* Participant characteristics

|  |  | Wave 1 |  | Wave 2 |  | Wave 3 |  |
| --- | --- | --- | --- | --- | --- | --- | --- |
|  | Units |  | N |  | N |  | N |
| Age | Yrs, M (SD) | 69.52 (0.83) | 1,060 | 72.49 (0.71) | 840 | 76.23 (0.67) | 675 |
| Sex | M:F | 523:537 | 1,060 | 428:412 | 840 | 343:332 | 675 |
| MMSE | M (SD) | 28.89 (1.25) | 1,059 | 28.83 (1.27) | 839 | 28.83 (1.30) | 675 |
| HADS | M (SD) | 7.67 (4.51) | 1,055 | 7.05 (4.40) | 839 | 7.47 (4.46) | 674 |
| BMI | M (SD) | 27.76 (4.36) | 1,058 | 27.90 (4.46) | 840 | 27.73 (4.50) | 672 |
| Hypertension | Y:N | 421:639 | 1,060 | 410:430 | 840 | 366:308 | 675 |
| Diabetes | Y:N | 85:975 | 1,060 | 89:751 | 840 | 76:599 | 675 |
| CVD | Y:N | 255:805 | 1,060 | 238:602 | 840 | 222:452 | 674 |
| Arthritis | Y:N | 466:593 | 1,059 | 386:454 | 840 | 313:361 | 674 |

*Note.* MMSE: mini mental state examination, HADS: hospital anxiety and depression scale, BMI: body mass index, CVD: cardiovascular history.

*Table S2.* Structural equation model fit statistics for visuospatial ability.

| **Model** | ***χ* ^2^** | **df** | ***p*** | **RMSEA** | **CFI** | **TLI** | **SRMR** |
| --- | --- | --- | --- | --- | --- | --- | --- |
| Sleep Quality | 58.52 | 49 | 0.166 | 0.014 | 0.998 | 0.996 | 0.015 |
| Bedtime | 59.10 | 49 | 0.153 | 0.014 | 0.997 | 0.996 | 0.016 |
| Latency | 53.51 | 49 | 0.305 | 0.009 | 0.999 | 0.998 | 0.015 |
| Wake Time | 54.86 | 49 | 0.262 | 0.011 | 0.999 | 0.998 | 0.015 |
| Length night | 62.16 | 49 | 0.098 | 0.016 | 0.997 | 0.995 | 0.016 |
| Length day | 55.03 | 49 | 0.257 | 0.011 | 0.999 | 0.998 | 0.015 |

*Note.* RMSEA: root mean square error of approximation, CFI: comparative fit index, TLI: Tucker Lewis Index, SRMR: standardised root mean square residual.

*Table S3.* Structural equation model fit statistics for processing speed.

| **Model** | ***χ* ^2^** | **df** | ***p*** | **RMSEA** | **CFI** | **TLI** | **SRMR** |
| --- | --- | --- | --- | --- | --- | --- | --- |
| Sleep Quality | 167.30 | 86 | <0.001 | 0.030 | 0.988 | 0.982 | 0.027 |
| Bedtime | 161.07 | 86 | <0.001 | 0.029 | 0.989 | 0.983 | 0.027 |
| Latency | 163.82 | 86 | <0.001 | 0.029 | 0.988 | 0.983 | 0.026 |
| Wake Time | 163.19 | 86 | <0.001 | 0.029 | 0.988 | 0.983 | 0.027 |
| Length night | 157.12 | 86 | <0.001 | 0.028 | 0.989 | 0.984 | 0.026 |
| Length day | 172.47 | 86 | <0.001 | 0.031 | 0.987 | 0.981 | 0.027 |

*Note.* RMSEA: root mean square error of approximation, CFI: comparative fit index, TLI: Tucker Lewis Index, SRMR: standardised root mean square residual.

*Table S4.* Structural equation model fit statistics for memory.

| **Model** | ***χ* ^2^** | **df** | ***p*** | **RMSEA** | **CFI** | **TLI** | **SRMR** |
| --- | --- | --- | --- | --- | --- | --- | --- |
| Sleep Quality | 89.42 | 48 | <0.001 | 0.029 | 0.989 | 0.982 | 0.025 |
| Bedtime | 97.71 | 48 | <0.001 | 0.030 | 0.988 | 0.980 | 0.026 |
| Latency | 84.47 | 48 | <0.001 | 0.027 | 0.990 | 0.984 | 0.024 |
| Wake Time | 88.01 | 48 | <0.001 | 0.028 | 0.989 | 0.982 | 0.024 |
| Length night | 86.06 | 48 | <0.001 | 0.027 | 0.990 | 0.983 | 0.024 |
| Length day | 84.40 | 48 | <0.001 | 0.027 | 0.990 | 0.984 | 0.024 |

*Note.* RMSEA: root mean square error of approximation, CFI: comparative fit index, TLI: Tucker Lewis Index, SRMR: standardised root mean square residual.

*Table S5.* Illustrative post-hoc analysis: associations between daytime sleep duration and growth curve of digit symbol substitution tests.

| *TEST* | Est. | SE | *p* | Baseline M(SD) |
| --- | --- | --- | --- | --- |
| Digit Symbol | -0.533 | 0.174 | 0.002 | 56.96 (12.78) |

*Note.* Unstandardised regression coefficients, standard errors and p-values reported. Log daytime sleep duration (hours) and cognitive test scores at each wave were corrected for age, sex, and time-varying measures of anxiety and depression symptoms (HADS), BMI, hypertension, diabetes, cardiovascular history and arthritis. Model fit parameters were SRMR ≤ 0.024, CFI ≥ 0.983, TLI ≥ 0.972, RMSEA = 0.026.

*Table S6.* Associations between polygenic scores for chronotype and self-reported sleep characteristics

| *CHRONOTYPE* | P≤1 | P≤0.5 | P≤0.1 | P≤0.05 | P≤0.01 |
| --- | --- | --- | --- | --- | --- |
| Quality | -0.026 (0.521) | -0.028 (0.485) | -0.053 (0.194) | -0.058 (0.158) | -0.030 (0.462) |
| Bedtime | **-0.106 (0.009)** | **-0.104 (0.011)** | **-0.085 (0.038)** | **-0.082 (0.046)** | **-0.084 (0.039)** |
| Latency^†^ | 0.010 (0.817) | 0.004 (0.919) | 0.020 (0.649) | 0.025 (0.565) | 0.011 (0.807) |
| Risetime | **-0.089 (0.031)** | **-0.098 (0.018)** | **-0.102 (0.013)** | -0.064 (0.124) | -0.064 (0.114) |
| Length night | -0.027 (0.518) | -0.022 (0.587) | -0.062 (0.135) | -0.043 (0.305) | -0.011 (0.782) |
| Length day^†^ | **0.102 (0.012)** | **0.109 (0.008)** | **0.123 (0.003)** | **0.090 (0.030)** | 0.032 (0.430) |

*Note.* Standardised regression coefficients (p-values) reported. Bold typeface denotes nominal significance (*p* < 0.05). No associations survived FDR correction for multiple comparisons.

*Table S7.* Associations between polygenic scores for duration and self-reported sleep characteristics

| *DURATION* | P≤1 | P≤0.5 | P≤0.1 | P≤0.05 | P≤0.01 |
| --- | --- | --- | --- | --- | --- |
| Quality | 0.033 (0.446) | 0.033 (0.437) | 0.023 (0.583) | 0.073 (0.084) | 0.030 (0.476) |
| Bedtime | -0.008 (0.846) | -0.014 (0.753) | -0.028 (0.512) | -0.004 (0.926) | 0.015 (0.730) |
| Latency^†^ | -0.060 (0.186) | -0.066 (0.148) | -0.04 (0.357) | **-0.101 (0.027)** | **-0.093 (0.043)** |
| Risetime | -0.013 (0.770) | -0.019 (0.664) | 0.002 (0.967) | -0.018 (0.664) | 0.012 (0.787) |
| Length night | **0.097 (0.025)** | **0.096 (0.025)** | **0.103 (0.015)** | **0.109 (0.010)** | **0.104 (0.016)** |
| Length day^†^ | 0.047 (0.283) | 0.042 (0.336) | 0.003 (0.939) | -0.006 (0.885) | -0.019 (0.658) |

*Note.* Standardised regression coefficients (p-values) reported. Bold typeface denotes nominal significance (*p* < 0.05). No associations survived FDR correction for multiple comparisons.
